# Musculoskeletal Infection Reporting and Data System (MSKI-RADS): reviewed and explained

**DOI:** 10.1186/s13244-025-02185-1

**Published:** 2026-02-24

**Authors:** Angela He, Flavio Duarte Silva, Mina Guirguis, Erin F. Alaia, William B. Morrison, Avneesh Chhabra

**Affiliations:** 1 Department of Radiology, UT Southwestern Medical Center, Dallas, TX USA; 2 Department of Radiology, NYU Langone Medical Center, New York, NY USA; 3 Department of Radiology, Thomas Jefferson University Hospital, Philadelphia, PA USA; 4 Department of Orthopaedic Surgery, UT Southwestern Medical Center, Dallas, TX USA

**Keywords:** Musculoskeletal infection, Osteomyelitis, MRI, X-ray, MSKI-RADS

## Abstract

**Abstract:**

A standardized guideline and scoring system are recommended for the imaging evaluation of musculoskeletal infections on MR imaging. The Musculoskeletal Infection Reporting and Data System (MSKI-RADS) is a recently developed and validated classification system using MR imaging that can be used to classify the severity and extent of musculoskeletal infections, improve radiology-pathology concordance, and outline the corresponding management recommendations. This review article explains MSKI-RADS and discusses the different elements of this system in detail with a review of pertinent literature so that the readers can apply it in their practices. The work outlines the technical considerations for optimal MR imaging for evaluating various musculoskeletal infectious lesions, details the severity scales with examples of various conditions that fall under each class, and outlines related patient management recommendations. The readers can learn about the MSKI-RADS classification system and apply the gained information from this article to improve MRI interpretations in their practice and increase the effectiveness of their multidisciplinary communications. This standardized system will also allow longitudinal data collection and tracking for future research purposes.

**Critical relevance statement:**

MSKI-RADS is a recently developed and validated MRI-based guideline for musculoskeletal infections in extremities. A comprehensive understanding of these classifications can facilitate improved standardized diagnostic reporting of musculoskeletal infections on MRI and better patient outcomes.

**Key Points:**

- Current terminology for describing musculoskeletal infections on MRI is nonspecific, resulting in confusing diagnostic reports.
- A standardized guideline and scoring system are critical for improving diagnostic reporting of musculoskeletal infections on MRI.
- MSKI-RADS is a recently developed and validated MRI-based guideline that can be used to characterize musculoskeletal infections in extremities.
- MSKI-RADS is a meaningful tool that facilitates improvements in standardized reporting and treatment protocols, multidisciplinary communications, and longitudinal data collection.

**Graphical Abstract:**

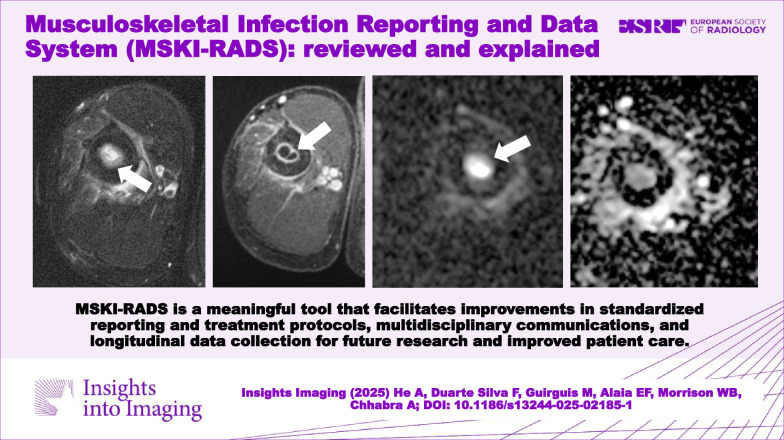

## Introduction

Musculoskeletal (MSK) infections encompass a broad spectrum of commonly encountered pathologies, including soft tissue infections, septic arthritis, and osteomyelitis. Of these conditions, osteomyelitis (OM) is a particularly serious infection that is important for radiologists to recognize and diagnose promptly. Osteomyelitis is an inflammatory destruction of the bone, commonly caused by bacterial infectious agents, such as *Staphylococcus aureus*. The pathogenesis of osseous infection can be due to direct inoculation, hematogenous seeding, or contiguous spread of microbial organisms [1, 2]. The annual incidence of OM in the United States has been estimated to be 21.8 cases per 100,000, with the prevalence increasing yearly [3]. The increase in OM prevalence can partly be attributed to the expanding older population as well as the increasing prevalence of predisposing conditions such as diabetes mellitus, malignancy, and immunocompromised status [1, 3, 4]. Increasing numbers of image-guided diagnostic and therapeutic procedures have also contributed to this reported increase in prevalence. Treatments used for OM, including long-term antibiotic therapy and surgical interventions, serve as the main drivers of cost, with diabetic foot osteomyelitis (DFO) management costing Medicare up to $13 billion annually [5, 6]. Therefore, prompt and accurate diagnosis of OM is critical in delivering timely care to patients and mitigating the need for more aggressive surgical interventions, thus improving overall patient outcomes and quality of life.

Obtaining adequate tissue samples, bacterial cultures, and/or bone biopsies to definitively diagnose OM can be challenging, of low yield, and onerous. Therefore, imaging plays a critical role in characterizing the location and severity of OM [7–9]. The American College of Radiology (ACR) Appropriateness Criteria recommends radiographs (XRs) as an initial screening modality for patients suspected of extremity OM and magnetic resonance imaging (MRI) as the examination of choice for confirming and delineating the extent of deep tissue involvement [10, 11]. MRI is extremely useful for musculoskeletal infection assessment due to its high diagnostic performance [12–14]. Decreased marrow signal intensity on T1W images in a medullary pattern and occurring with confluent distribution as a diagnostic marker of OM has high sensitivity, specificity, and average accuracy [15].

While MRI has many advantages, rendering a confident and clinically useful OM diagnosis with MRI for effective multidisciplinary care requires reader expertise and a standardized reporting system. Current terminology for describing findings of musculoskeletal infections, such as OM on MRI, has largely been adopted from the terminology used for radiographs and is nonspecific and inconsistent when applied to MRI reporting, resulting in confusing and nonspecific radiology reports that may provide little aid in clinical management and decision-making [7, 16]. To address this, Alaia et al proposed a standardized nomenclature for describing MRI findings of musculoskeletal infections in extremities in a Society of Skeletal Radiology white paper [7].

Recently, a multidisciplinary team developed and validated an MRI-based guideline, titled “MSKI-RADS: an MRI-based Musculoskeletal Infection Reporting and Data System for the Diagnosis of Extremity Infections” [17, 18]. This review article describes the MSKI-RADS guideline and expands the details with illustrative examples to aid the readers in understanding the scoring system and its clinical application in their practices. Together with the availability of clinical history and examination findings, MSKI-RADS can serve as a meaningful asset for improvements in standardized reporting and treatment protocols, multidisciplinary communications, and longitudinal data collection for future research and improved patient care.

## Technical considerations of MSK infection MR imaging

MR imaging of the extremities is ideally obtained on high-field 3-T or 1.5-T scanners. Multi-channel flexible surface coils or joint-specific coils aid in producing a high signal-to-noise ratio and superior contrast imaging. 3.0-T imaging is generally preferred over 1.5-T for most MSK MRI examinations due to the desired higher resolution of small structures in question, except in patients with indwelling hardware, which causes more susceptibility artifacts as the strength of the magnetic field increases [19]. However, this has become less important due to the development of more sophisticated artifact-reducing imaging protocols [20].

The imaging protocols should include T1-weighted fast spin echo (T1W FSE) and fluid-sensitive sequences, either short tau inversion recovery (STIR) images, T2-weighted FSE with fat saturation (T2W FS) or proton density-weighted FSE images with fat saturation (PDW FS) using frequency selective / Dixon protocols. Fat suppression on fluid-sensitive sequences is an important step in musculoskeletal infection imaging, and STIR offers superior fat saturation over chemical shift techniques in the following scenarios: the target body part demands a larger field-of-view, regional metal is present, imaging is off-center and challenging anatomic regions (multiple air tissue interfaces) are being imaged. This is because STIR is insensitive to B0 and B1 heterogeneity [21]. The suggested protocol used at our institution is outlined in Table 1. Ideally, isotropic small voxel (~1.5–2.0 mm) 3D gradient echo T1W imaging with fat suppression is obtained on high-field scanners before and after the intravenous administration of contrast and reformatted in multiple planes [17, 22]. Alternatively, non-fat-suppressed images can be obtained pre- and post-contrast, followed by subtraction manipulation. Contrast-enhanced imaging is helpful in multiple ways. In the author’s opinion, it increases the diagnostic confidence of infectious osteomyelitis as more intense enhancement is seen in the cases of infected bone versus reactive noninfectious inflammation of bone. Contrast is better in delineating the deeper extent of infectious soft tissue involvement (differentiates non-enhancing bland edema from enhancing cellulitis), characterizing the presence of abscesses, outlining the sinus tract and devitalized tissues, and aiding in the detection of soft tissue or intra-osseous gas and foreign bodies [23]. Contrast imaging may be contraindicated in patients with prior severe allergic reaction to gadolinium-based contrast agents and those with very poor and rapidly deteriorating renal function (glomerular filtration rate (GFR) < 15–30 mL/min). In such circumstances or otherwise, diffusion-weighted imaging (DWI) and apparent diffusion coefficient (ADC) maps can help to identify bone or soft tissue abscesses [22, 24]. DWI has been found to be useful in differentiating purulent material in localized soft tissue or intra-osseous abscess (ADC = 0.5–0.9 × 10^−^^3^ mm^2^/s) from bland non-purulent fluid collections. In communicating abscess with a fistulous tract, only margins of the abscess usually restrict. In addition, DWI can help detect a ‘ghost sign’ on ADC maps with the disappearance of the OM bone as compared to non-infected bone marrow edema. DWI is more sensitive than T1W imaging for the detection of a ghost sign [25]. In septic arthritis, due to excessive joint fluid, only mild restriction of ADC (ADC = 1.7–2.3 × 10^−^^3^ mm^2^/s) is usually apparent unless there is intra-articular localized purulent material. Finally, DWI has also been found to be useful in differentiating Charcot neuroarthropathy from OM [26, 27].

**Table 1:** Musculoskeletal extremity infection MRI protocol

MRI sequence	2D/3D	Section thickness/gap (mm)	Pixel size (F x P) (mm)	TR (repetition time) (ms)	TE (echo time) (ms)	Other
Three-plane Scout	2D	5.0		8.0	4.0	
Coronal T2-weighted DIXON	2D	4.0/0.4	0.5 × 0.6	3000–4500	40–45	
Sagittal STIR	2D	4.0/0.4	0.5 × 0.6	3000–4500	30–35	
Sagittal T1-weighted TSE	2D	4.0/0.4	0.5 × 0.6	450–600	6–9	
Axial T2-weighted DIXON	2D	3.0–4.0/0.4	0.5 × 0.6	3000–4500	40–45	
Axial T1-weighted TSE	2D	3.0–4.0/0.4	0.5 × 0.6	450–600	6–9	
Axial T1-weighted DIXON or VIBE PRE	3D	1.5 ISO/0	Acquired ISO	Lowest	Lowest	
Axial T1-weighted DIXON or VIBE POST	3D	1.5	Acquired ISO	Lowest	Lowest	Isotropic
Axial DWI	2D	4.0/0	2 × 2	2500–4500	65–70	B – 50, 400, 800 ADC map sent to PACS

*2D* two-dimensional, *3D* three-dimensional, *TSE* turbo spin echo, *VIBE* volumetric interpolated breath-hold examination, *DWI* diffusion-weighted imaging, *STIR* short tau inversion recovery, *PRE* pre-contrast, *POST* post-contrast, *ADC* apparent diffusion coefficient

A standardized lexicon is recommended to describe the terms for musculoskeletal infections.

## Lexicon with recommended MRI terminology for MSK infectious pathology description

It is prudent to use appropriate terms to describe the extent and type of infections. The following discussion focuses on recommended terms and lexicon to use in reporting MSK infections.

### Ulcer

An ulcer is defined as a focal discontinuity of the skin surface at the site of suspected MSK infection. It is commonly associated with cellulitis (see below) and may have a sinus tract involving deeper tissues (subcutaneous, fascia, muscle, or bone). A fistulous tract denotes tubular bidirectional communication from the ulcer site to the deeper abscess/collection.

### Cellulitis

Bland edema is seen as an increased signal on T2W or fluid-sensitive images with or without decreased T1W signal alteration. It can be focal or diffuse and may be seen as a result of local injury, cardiac decompensation, or lymphedema, among many other causes. Cellulitis refers to a superficial soft tissue infection affecting the skin and subcutaneous tissues without extension to the deep fascia, presenting on MRI as skin and/or subcutaneous thickening with increased signal on fluid-sensitive sequences and correspondingly low T1 signal. Due to inflammation and increased vascular permeability, varying degrees of contrast enhancement are seen with cellulitis. Superficial perifascial edema at the deeper interface with the subcutaneous edema might be present; however, underlying unaffected muscles show normal intermediate signal intensity. Cellulitis can be focal or diffuse and is more likely in the event of an ulcer. It is best to use the term focal cellulitis rather than a nonspecific term such as a phlegmon or phlegmonous change.

### Abscess

An abscess is a walled-off fluid collection that typically manifests as hypo- or iso- to slightly hyperintense T1W signal compared to the skeletal muscle, with signal intensity usually higher at the periphery, and heterogeneous and intense internal T2W hyperintensity or fluid level. They are also often surrounded by soft tissue edema. Abscesses can be unifocal or multifocal, arising in any of the superficial tissues, deeper soft tissues, or bone (intra-osseous abscess) [28, 29]. Typically, abscesses on contrast-enhanced MRI show early and persistent intense peripheral or internal septal enhancement. Hopkins et al found the presence of an enhancing rim on post-gadolinium-DPTA MRI to have a sensitivity of 89% and a specificity of 80% in diagnosing soft tissue abscesses [30]. Surrounding hyperemic or necrotic non-enhancing tissues may be observed as well. ADC restriction is typical in the core or lining of abscesses with low ADC values of 0.5–1.1 × 10^−^^3^ mm^2^/s, with even higher ADC values in the core of the externally communicating abscess. Although hematomas can also restrict ADC and show peripheral enhancement, additional imaging findings in the setting of suspected infection, e.g., regional cellulitis, skin ulceration or a sinus tract, inner hyperintense rim on T1W images, air-fluid level, etc., aid in rendering an appropriate diagnosis of an abscess.

### Non-necrotizing and necrotizing fasciitis

Both these entities constitute an infection that involves deeper fascia. Clinically, necrotizing fasciitis (NF) is a rapidly progressive infectious process with a high mortality rate despite timely treatment and is a surgical emergency [31]. The definite diagnostic criterion to distinguish NF from non-NF is surgical exploration that demonstrates necrotic fat with a brownish color and lack of resistance to manual debridement along the deep fascial planes [32]. According to Kim et al, MRI findings useful in distinguishing NF from non-NF include: (1) thickening and T2W hyperintensity of the deep fascia measuring 3.0 mm or more in thickness, especially when extending through the deep intermuscular fascia distant from the deep peripheral fascia; (2) multicompartmental involvement; (3) focal or diffuse absence of post-contrast enhancement along the fascia and perifascial soft tissues (26% of non-NF and in 86% of NF); (4) low signal intensity foci present on all sequences that suggest soft tissue emphysema (present in less than 50% of NF) [32–34].

### Septic arthritis

Intra-articular contiguous or hematogenous spread of infection into the joint space results in septic arthritis. MR imaging is extremely sensitive in detecting joint effusions, even very small ones, making it a useful aid in the diagnosis of septic arthritis [28]. Hopkins et al found that the sensitivity and specificity of gadolinium contrast-enhanced MRI with fat suppression were 100% and 77%, respectively, for detecting septic arthritis [30]. On MRI, significant joint effusion with or without complex debris/air, synovial hypertrophy, marked synovitis with synovial enhancement (lamellated, frond-like), and perisynovial/pericapsular and regional fascia edema are key findings that suggest septic arthritis over inflammatory arthritis, which can present with overlapping findings on imaging [35]. According to Lee et al, signal abnormalities in adjacent bone marrow on fat-suppressed gadolinium-DPTA enhanced images can also help differentiate septic arthritis from other similar entities, particularly transient synovitis [36]. It should be noted that septic arthritis may also occur without substantial joint effusion. Regional cellulitis and other clinical and radiological signs of infection, including osteomyelitis, aid in rendering the diagnosis of septic arthritis. Additionally, diffusion restriction can assist in distinguishing purulent material within the joint from non-purulent inflammatory arthritis.

### Infectious bursitis and infectious tenosynovitis

Bursitis can be caused by sterile inflammation of the bursa. Direct inoculation or hematogenous spread of organisms can also lead to infection of bursa (congenital, e.g., subacromial-subdeltoid or acquired, adventitial bursa over friction sites) or a tendon sheath. As tendon sheaths and bursa both contain synovial epithelium, manifestations of tenosynovitis and bursitis can resemble a joint infection radiologically [37]. On MRI, infectious bursitis or tenosynovitis may show variable amounts of intra-bursal or tendon sheath fluid present, often with extensive peribursal or peri-tendon sheath edema and internal septations/debris. Like an abscess, infected bursitis and tenosynovitis can present with minimal T1W hyperintensity, diffusion restriction, and intense peripheral and/or septal enhancement [38].

### Osteomyelitis (OM)

Osteomyelitis involves infection of the bone and bone marrow with marrow edema, cellular infiltration, vascular engorgement, and bone necrosis. It may also be complicated by the formation of an abscess. In the setting of OM, complicating abscesses can develop in the intramedullary cavity, between the cortex and the periosteum, and/or may extend into the adjacent soft tissues. In chronic osteomyelitis, the loss of vascular supply to an area of bone may result in the formation of a necrotic, devitalized bone fragment, known as a sequestrum, that is surrounded by granulation tissue. An involucrum, which is a thick sheath of periosteal new bone, can also develop around the sequestrum. An opening, or cloaca, may then form in the involucrum, allowing necrotic bone and debris to drain.

MR imaging is a very important diagnostic tool for osteomyelitis, given its anatomic detail and disease characterization. According to the literature, the sensitivity and specificity of MRI for detecting OM range from 86 to 98% and 75 to 100%, respectively, as reported in various small case series [28, 39]. On MRI, confluent low T1W signal, loss of cortical hypointensity from cortical erosions, and often more extensive hyperintense signals on STIR/T2FS are the hallmarks of this disease [40]. OM is also often accompanied by periostitis, cortical bone enhancement, intra-osseous abscess and regional cellulitis, fasciitis, and/or septic arthritis. Of note, the usage of terms including osteitis, which is commonly defined as infectious involvement of the cortical bone, or reactive bone marrow edema to describe discordant marrow signal changes on MRI in cases of suspected OM, should be avoided due to their misleading nature that may prompt incorrect management [7].

MRI is also helpful for distinguishing between acute and chronic osteomyelitis. In acute osteomyelitis, edema and soft tissue infiltration associated with the acuity of the disease process make it difficult to appreciate the interface between normal and abnormal bone marrow. Cortical thickening would also not be observed. On the other hand, in chronic osteomyelitis, devascularized and fibrotic areas of bone sequestration in the marrow can be seen as low signal intensity areas on both T1-weighted and T2-weighted images. Contrast enhancement further helps with differentiation, as areas of devitalized sequestration do not enhance, whereas areas of active inflammation in soft tissue and bone marrow do [28].

## MSKI-RADS lexicon for MSK infectious pathology

The following RADS categories outline the severity scoring system in MRI-based criteria for the diagnosis of MSK infections.

MSKI-RADS 0 refers to a nondiagnostic or incomplete study in which the interpreting radiologist suggests that the imaging quality of the examination is inadequate or hinders the identification of findings or diagnosis.

MSKI-RADS I (negative for infection) refers to a study in which the interpreting radiologist suggests no MSK infection is identified (Supplementary Fig. 1). Findings may include bland subcutaneous soft tissue edema with suggestive diagnoses of lymphedema, scarring, morphea, or cardiac failure, etc.

**Fig. 1:**
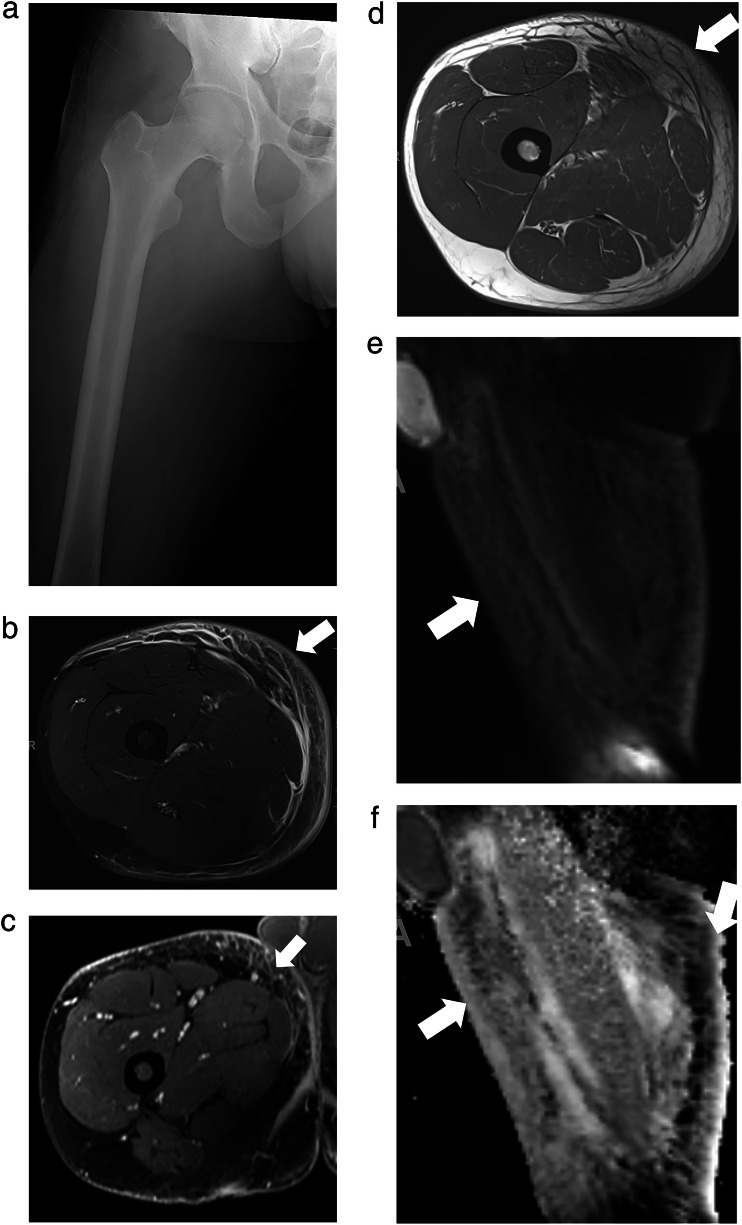
MSKI-RADS Class II. A 35-year-old man with right thigh pain and swelling. **a**–**d** Frontal X-ray, Axial T1W and fsT2W (water map Dixon), and axial 3D fsT1W post-contrast images, respectively, of right thigh showing soft tissue edema, skin thickening and cellulitis (arrows) consistent with superficial soft tissue infection (class II). **e**, **f** Corresponding DWI and ADC sagittal maps confirm cellulitis with no significant diffusion restriction to suggest underlying abscess

MSKI-RADS II (superficial soft tissue infection) refers to a cutaneous-subcutaneous lesion identified with enhancement on contrast-enhanced imaging. This class includes diagnoses such as infected ulcer, cellulitis, or cutaneous abscess/furuncle that can be accessed with a superficial approach in the doctor’s office and emergency room (Fig. 1).

**Fig. 2:**
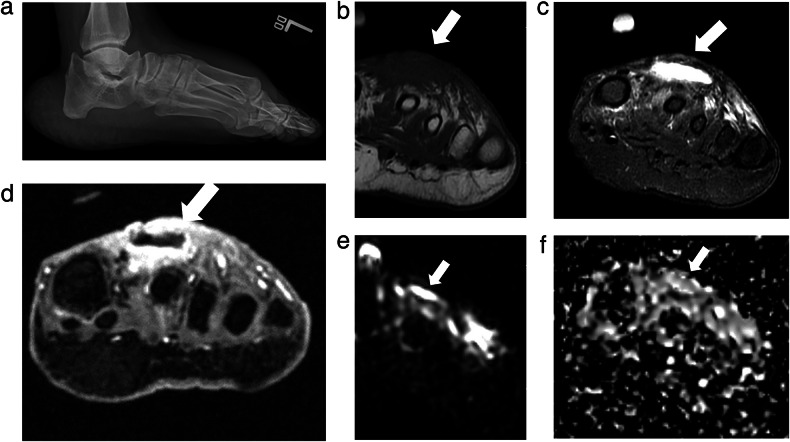
MSKI-RADS Class III. A 35-year-old man with foot infection. **a** Lateral X-ray of the affected left foot. **b**–**d** Axial T1W, fsT2W (water map Dixon), and 3D T1W axial post-contrast images of the left foot showing a peripherally enhancing collection (arrows), reflecting a subcutaneous abscess consistent with deeper soft tissue infection (class III). **e**, **f** Corresponding DWI and ADC map of the left foot show mild diffusion restriction of the area of cellulitis and abscess

MSKI-RADS III (deeper soft tissue infection) refers to the identification of a deeper subfascial abscess (Fig. 2)/inter-fascial and/or intramuscular infectious lesion or deeper abscess that may require a surgeon to access them in the operating room.

**Fig. 3:**
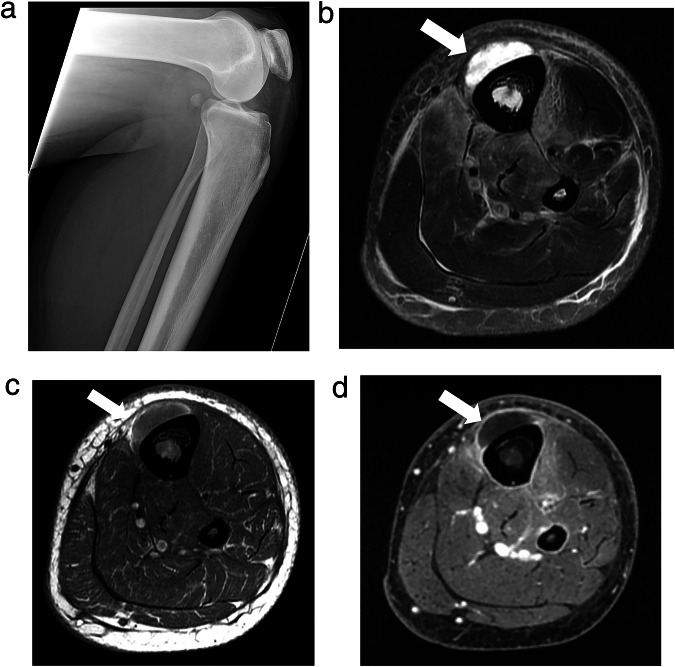
MSKI-RADS Class IV. A 21-year-old woman with left lower leg infection. **a** Lateral X-ray of the affected left lower leg. **b**–**d** Axial fsT2W (water map Dixon), T1W, and axial 3D T1W post-contrast images, respectively showing subperiosteal abscess (arrows) of anterior tibia without confluent T1 marrow signal alteration or enhancement consistent with possible osteomyelitis. The surgical incision, drainage, and biopsy showed fibrous reactive synovial type tissue of the periosteum with patchy chronic inflammation. Bone fragments of the biopsy showed fewer than 2 neutrophils per high-power field

MSKI-RADS IV refers to findings that suggest possible osteomyelitis when no definitive findings for osteomyelitis have been identified. For example, a lack of confluent T1W signal hypointensity or cortical erosion with the presence of intra-osseous bone edema at the site of ulcer (Fig. 3). Instead of using nonspecific terms of reactive osteitis, osteitis, reactive bone marrow edema, etc., it is prudent to use category IV in the impression of the radiology reports when summarizing the findings. Terms like reactive osteitis are ambiguous, and the treating physician can be baffled as to whether they should be treated as OM or a soft tissue infection. It has been shown that more than 50% of such cases ultimately lead to frank OM [40]. Thus, using a uniform score based on imaging findings is helpful to guide management.

**Fig. 4:**
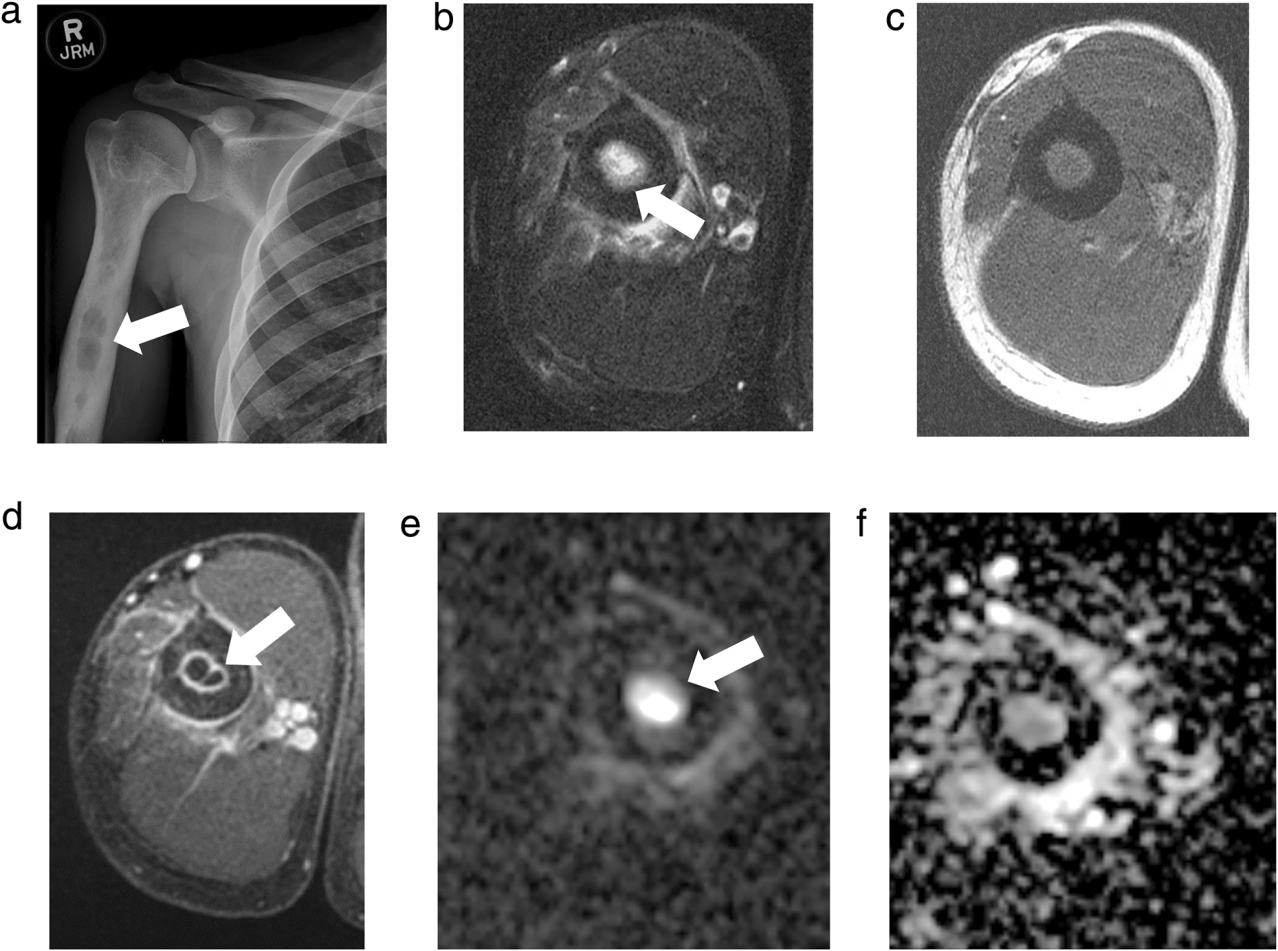
MSKI-RADS Class Va. A 30-year-old man with right arm infection. **a** Frontal X-ray of the right humerus showing an irregular lytic lesion (arrow), concerning for an intra-osseous abscess. **b**–**d** Axial fsT2W (Dixon water map), T1W, and 3D T1W post-contrast images, respectively, of right humerus showing peripherally enhancing intra-osseous abscess (arrows), consistent with subclass Va. **e**, **f** Corresponding DWI and ADC map of right humerus showing the associated diffusion restriction

MSKI-RADS V (definitive OM and/or septic arthritis) is divided into three subcategories: MSKI-RADS Va refers to a lesion that is highly likely to be osteomyelitis (Fig. 4). MSKI-RADS Vb refers to findings suggesting a high likelihood of septic arthritis. MSKI-RADS Vc refers to findings suggesting the high likelihood of both osteomyelitis and septic arthritis being present (Supplementary Fig. 2).

**Fig. 5:**
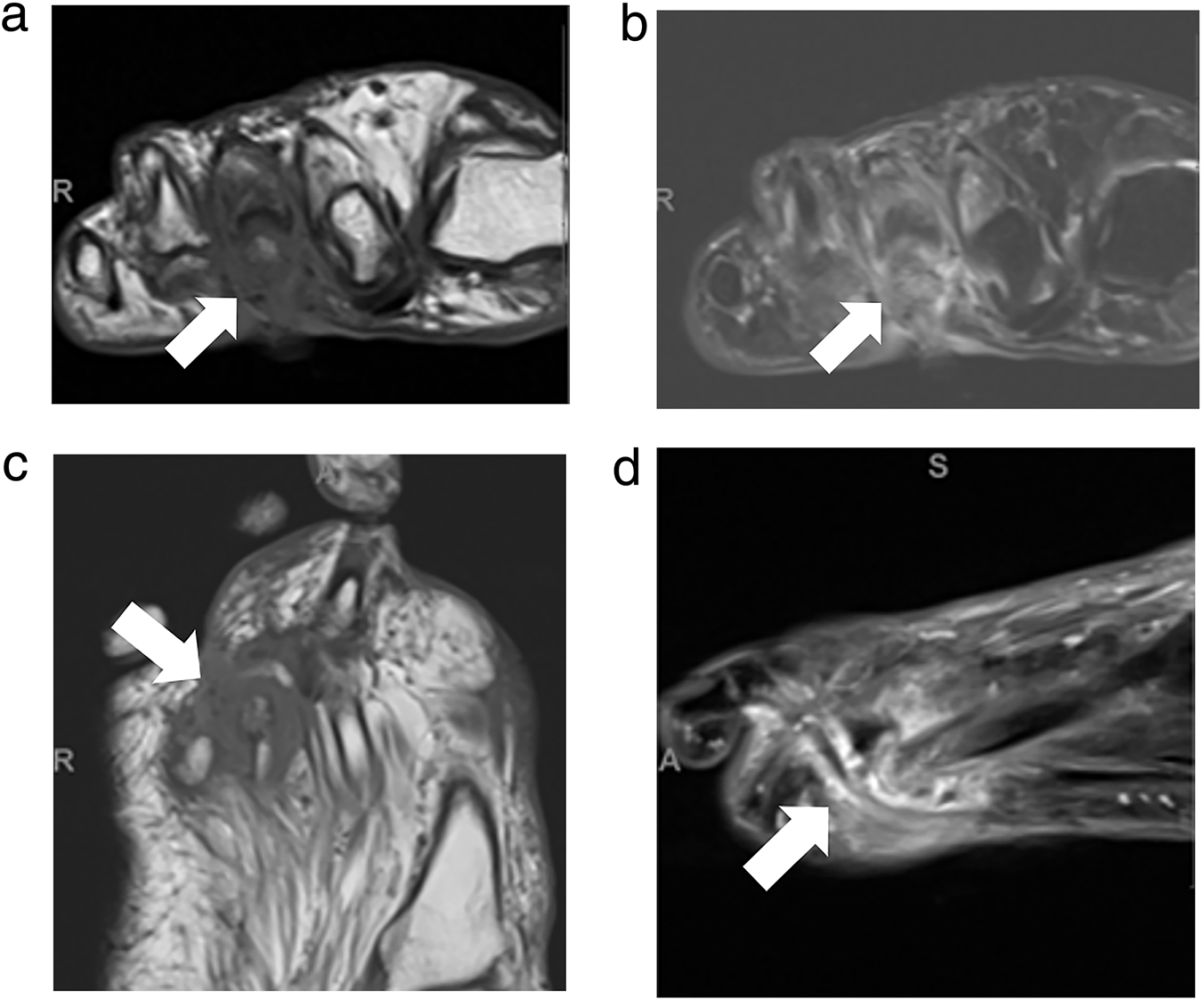
MSKI-RADS VIc. A 70-year-old woman with known osteomyelitis and septic arthritis of the 3rd metatarsophalangeal joint of the right foot, previously treated with antibiotic treatment. **a**–**d** Axial T1W, fsT2W (water map Dixon), coronal T1W and sagittal fsT2W (water map Dixon) images, respectively, of the right foot showing persistent plantar ulceration with cellulitis, synovitis, and bone destruction (arrows) in keeping with worsening osteomyelitis and septic arthritis following 4 weeks of antibiotic treatment, consistent with MSKI-RADS Class VIc

MSKI-RADS VI refers to studies in which the patient is undergoing or has recently undergone treatment for known or presumed osteomyelitis and/or septic arthritis. MSKI-RADS VI is divided into three subclassifications: MSKI-RADS VIa refers to findings in which no residual infection is identified, e.g., lack of bone marrow edema and/or normal fatty marrow replacement. MSKI-RADS VIb refers to the identification of a possibly persistent infection with persistent bone marrow edema and findings of previously seen infection. MSKI-RADS VIc refers to the identification of definitely persistent and/or worsening infection (Fig. 5). New areas of regional infection also fall into this category.

**Fig. 6:**
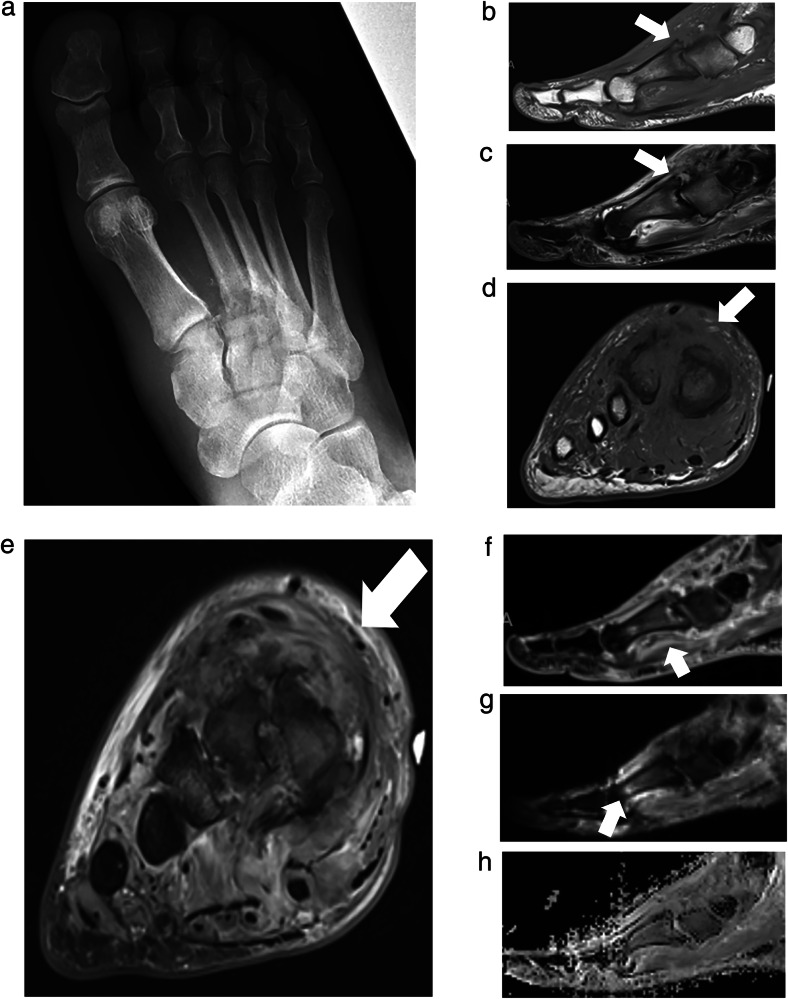
MSKI-RADS NOS. A 54-year-old man with known DM and mid-foot swelling. **a** Frontal X-ray of the foot. **b**, **c** Sagittal T1W and STIR images show 1st tarsometatarsal subchondral erosions and dorsal subluxation with regional soft tissue swelling and edema (arrows). **d**, **e** Axial T1W and fsT2W (water map Dixon) images show 1–3rd metatarsal bony cortical erosions and soft tissue swelling (arrows). **f** Sagittal fsT1W 3D image shows mild bone marrow and diffuse soft tissue enhancement (arrow) without soft tissue ulcer or abscess. **g**, **h** Corresponding sagittal DWI and ADC maps show mild T2-shine through of edema of the 1st tarsometatarsal articulation without a restricting abscess (arrow). Findings are consistent with Charcot neuroarthropathy-MSKI-RADS-NOS class

MSKI-RADS NOS (OM mimics) refers to the identification of nonspecific bony MRI changes, suggesting noninfectious etiology, including inflammatory arthropathy, gout, and Charcot neuroarthropathy (CNA), stress injuries, non-bacterial OM, chronic recurrent multifocal OM, etc. (Fig. 6). If there is a patient with Charcot neuroarthropathy (CNA) with coexistent infection, it receives a MSKI-RADS score of II-V versus NOS for isolated CNA without infection. The CNA findings can be described in the body of the report as such.

Importantly, the highest class is always used for the benefit of patient management, and multiple classes are not assigned to a particular case. For example, if a patient has osteomyelitis with fasciitis and cellulitis, it would be class V and not class II, III, and V.

In a recent multicenter and multi-institutional validation study using 20 MSK-fellowship fellowship-trained readers from multiple institutions, this system showed higher accuracy over qualitative diagnosis rendered by the readers (*p* < 0.05) and moderate inter-reader reliability (ICC = 0.7). In addition, high true positive rates were seen for cases negative for infection (MSKI-RADS I and NOS) at 88.7% (95% CI: 84.6%, 91.7%) and for MSKI-RADS V (highly suggestive of OM and/or septic arthritis) at 72.6% (95% CI: 63.4%, 80.1%), The lowest true positive rate was seen for MSKI-RADS IV (possible OM), which is likely related to a well-known pitfall of MRI interpretations and has been described using multiple terms, such as reactive edema and reactive osteitis. Many of these foot and ankle cases falling under MSKI-RADS IV eventually develop OM and thus should receive close follow-up [17].

## MSKI-RADS management protocols

The recommended management and treatment protocols for the proposed MSKI-RADS system were also developed in collaboration with a multidisciplinary team to assist in timely and appropriate patient treatments aiming at improved outcomes.

**MSKI-RADS 0** (nondiagnostic study or incomplete MR imaging)—the patient should be recalled for additional imaging, e.g., for missing sequence, or intravenous contrast scan, etc.

**MSKI-RADS I** (negative for infection)—for such patients, no further imaging for infection is recommended unless clinical worsening occurs over time, and repeat imaging is strongly considered clinically.

**MSKI-RADS II** (superficial soft tissue infection)—medical treatment includes a course of antibiotics and/or anti-inflammatory drugs. Further follow-up XR or MR imaging may be performed as per the recommendations of the clinical team.

**MSKI-RADS III** (deeper soft tissue infection)—as with MSKI-RADS II studies, recommended medical treatment involves a course of antibiotics and/or anti-inflammatory drugs. Additional treatment considerations include a biopsy of the lesion or surgical drainage of the collection and/or tissue debridement, followed by culture/sensitivity analysis of the specimen.

**MSKI-RADS IV** (possible OM)—the patients are recommended to receive a course of antibiotics and anti-inflammatory drugs. The clinical team should consider a bone biopsy and/or tissue debridement with culture/sensitivity analysis of the collected sample. Follow-up MR imaging may be considered as per the recommendations of the clinical team.

**MSKI-RADS V** (highly suggestive of OM)—medical treatment is recommended to include a course of antibiotics and anti-inflammatory drugs. The clinical team may consider a bone biopsy or surgical intervention, joint wash-out for suspected septic arthritis, and/or tissue debridement followed by culture/sensitivity testing of the collected sample, depending on the site(s) of infection. MR imaging follow-up in 6–8 weeks post-treatment is also recommended to assess improvement/resolution and as a guide for further management, e.g., rescoring, change of antibiotics, further surgical intervention, etc.

**MSKI-RADS VI** (known or presumed OM and/or septic arthritis on treatment)—as such treatment recommendations depend on the subclassification. For VIa cases, no further follow-up imaging for infection is recommended. For VIb, cases depicting possible persistent infection, the clinical team is suggested to consider follow-up MR imaging in 6–8 weeks after continued treatment. For studies depicting persistent or worsening infection that are categorized as VIc, recommended medical treatment involves a course of antibiotics and anti-inflammatory drugs. The patient may also need to modify the antibiotics depending on the results of the culture/sensitivity analysis. Additionally, the clinical team should consider bone biopsy or surgical intervention, joint wash-out for suspected septic arthritis, and/or tissue debridement followed by culture/sensitivity testing, depending upon the site(s) of infection involvement. Consider follow-up MR imaging in 6–8 weeks post-treatment.

**MSKI-RADS NOS** (nonspecific noninfectious bony changes)—the clinical team may consider rheumatologic consultation, laboratory work, and follow-up XR or MR imaging as per the evolution of clinical findings.

In conclusion, by following this compartmental distribution of severity scoring using a standardized lexicon and suggested management guideline, the various physicians and mid-levels involved in the multidisciplinary care of musculoskeletal infections can communicate effectively. They can standardize the description of the infectious lesions to improve radiology-pathologic concordance and interdisciplinary communications for optimized and timely patient care.

There will be a learning curve for less experienced radiologists, particularly in differentiating between classes II and III, and classes IV and V, as well as gaining knowledge about the subcategories in their practices. The MSKI-RADS system, crafted with input from an interdisciplinary team including experts in infectious diseases and surgical interventions, outlines treatment protocols, thus providing clear treatment considerations for every member of the team. Future longitudinal multi-institutional studies may aid in validating this classification system among junior and general radiologists and further refine the treatment protocols for the described categories aimed at improving patient management and treatment outcomes.

## Conclusion

MSKI-RADS is a novel MRI-based classification system for scoring infectious pathologies of the musculoskeletal system. Current practices for reporting musculoskeletal infection findings on MRI are limited by nonstandard terminology and descriptions that provide little guidance in clinical management. Adoption of this system can facilitate standardized reporting and management protocols across health professions and multiple institutions. Potential barriers to the implementation of MSKI-RADS include the need for initial training, especially for general radiologists, any associated costs, and possible pushback from other healthcare professionals given the unfamiliarity of the system. However, the advantages that MSKI-RADS offers in terms of standardization and ease in learning the system regardless of experience make it an appealing reporting system that can be readily integrated into multiple institutions.

Collection of longitudinal data using this system may facilitate further refinement of the treatment protocols to improve patient outcomes. Further research directions include generalizability studies that evaluate how general radiologists and trainees perform using MSKI-RADS after some training, as well as prospective studies assessing the impact of the system in the broader clinical practice.

## Supplementary information

ELECTRONIC SUPPLEMENTARY MATERIAL

## Abbreviations

MSKI-RADS: Musculoskeletal Infection Reporting and Data System
OM: Osteomyelitis
