# Supplementary material for: Musculoskeletal Infection Reporting and Data System (MSKI-RADS): reviewed and explained
Source: Insights Imaging. 2026 Feb 24;17:53. doi: 10.1186/s13244-025-02185-1 (PMC12932762; doi:10.1186/s13244-025-02185-1)
Supplement: Supplementary file 1 — ELECTRONIC SUPPLEMENTARY MATERIAL [file 13244_2025_2185_MOESM1_ESM.pdf]

Musculoskeletal Infection Reporting and Data System (MSKI-RADS): reviewed and explained

ELECTRONIC SUPPLEMENTARY MATERIAL

Insights Imaging (2025) He A, Duarte Silva F, Guirguis M, Alaia EF, Morrison WB, Chhabra A.

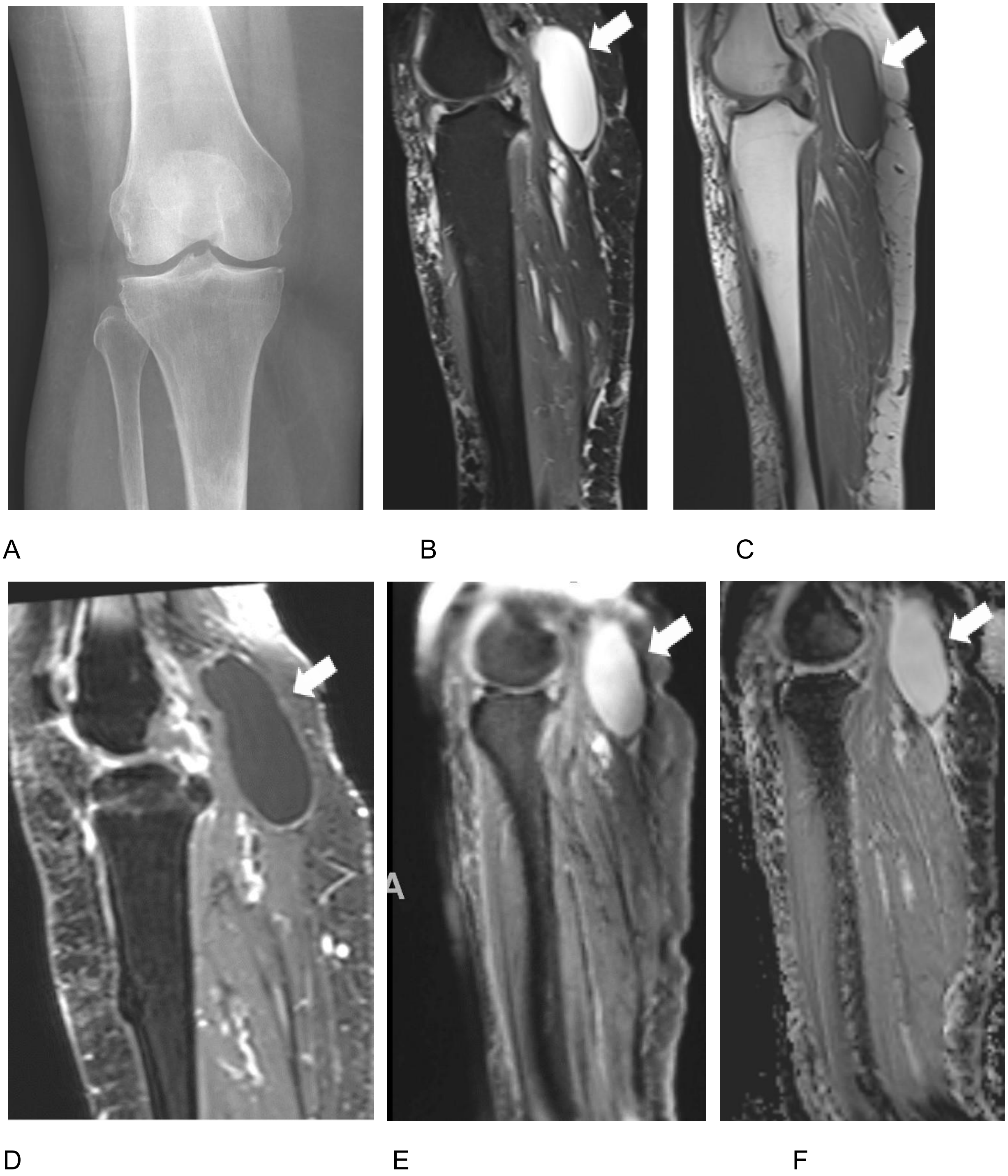

Supplementary Figure 1: Musculoskeletal Infection Reporting and Data System (MSKI-RADS) Class I. 55-year-old woman with knee swelling. A, B, C, D – Frontal X-ray, sagittal fsT2W (water map Dixon), T1W, and 3D T1W reconstructed post-contrast images, respectively, of the right knee and lower leg showing a Baker's cyst with peripheral enhancement but no signs of infection (arrows), consistent with MSKI-RADS Class I. E, F – Corresponding DWI and ADC images show no significant diffusion restriction.

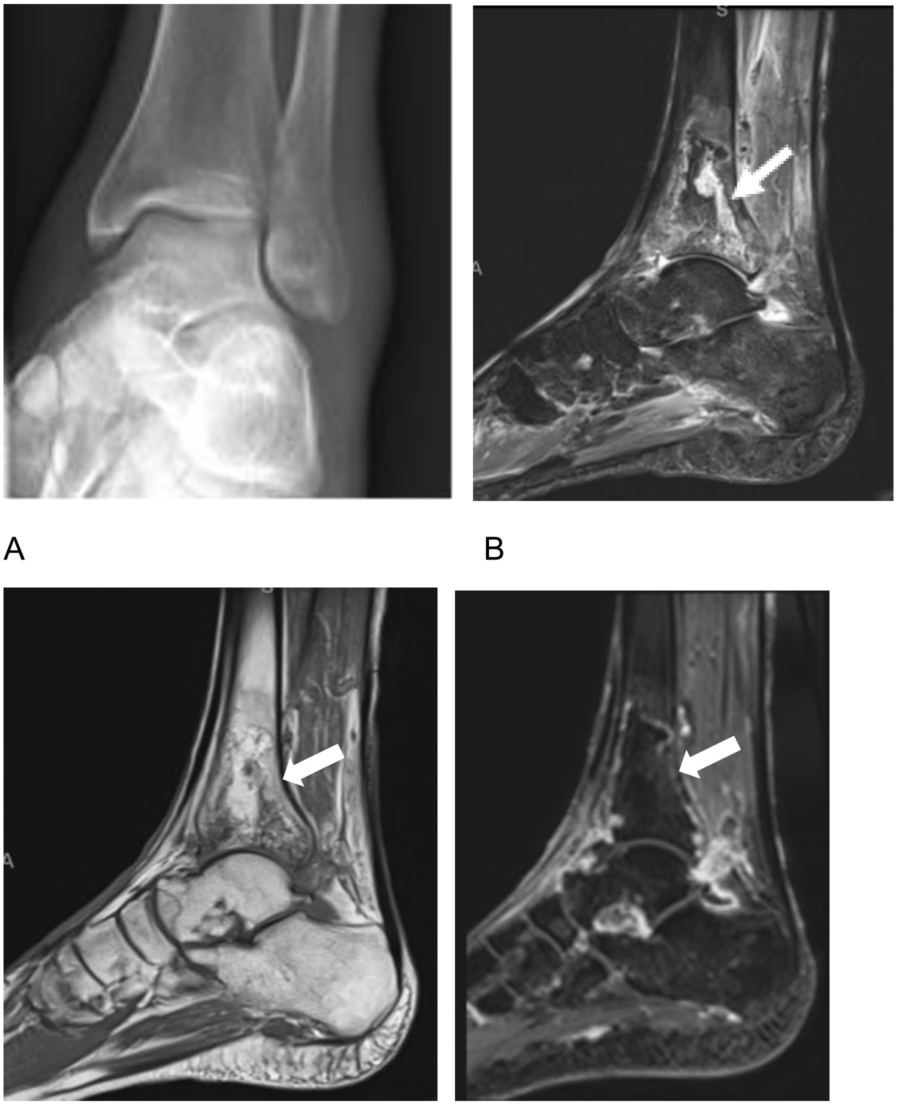

Insights Imaging (2025) He A, Duarte Silva F, Guirguis M, Alaia EF, Morrison WB, Chhabra A.

C D

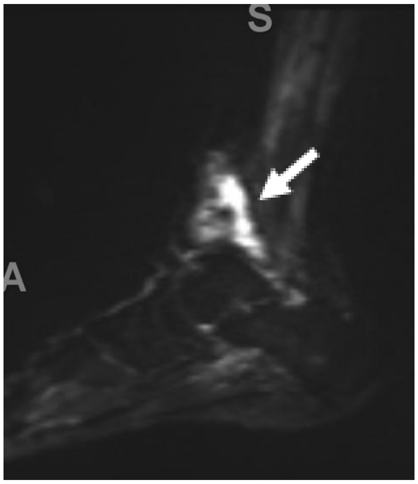

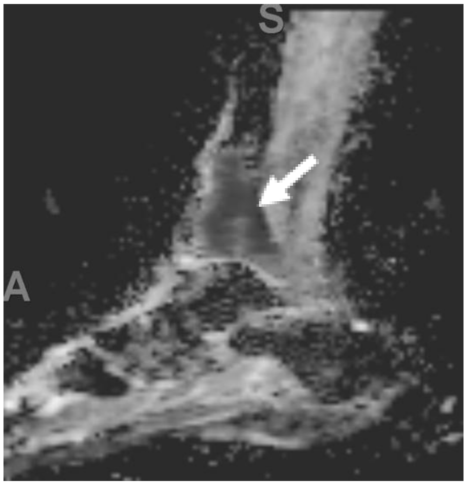

E F

Supplementary Figure 2: MSKI-RADS Vc. 65-year-old man with left ankle infection. A, B, C, D – Frontal x-ray, sagittal STIR, T1W, Dixon fs 3DT1W post-contrast images, respectively, of left ankle showing osteomyelitis with bone infarct, intraosseous distal tibial abscess (arrows), and tibiotalar synovitis consistent with septic arthritis and MSKI-RADS Class Vc. E, F – Corresponding DWI and ADC showing associated restriction diffusion (arrows).
